# Development of a risk score to identify patients at high risk for a severe course of COVID-19

**DOI:** 10.1007/s10389-023-01884-7

**Published:** 2023-03-22

**Authors:** Josephine Jacob, Falko Tesch, Danny Wende, Manuel Batram, Friedrich Loser, Oliver Weidinger, Martin Roessler, Martin Seifert, Lisa Risch, Oliver Nagel, Christina König, Roland Jucknewitz, Marina Treskova-Schwarzbach, Dagmar Hertle, Stefan Scholz, Stefan Stern, Pedro Ballesteros, Stefan Baßler, Barbara Bertele, Uwe Repschläger, Nico Richter, Cordula Riederer, Franziska Sobik, Anja Schramm, Claudia Schulte, Jochen Walker, Jochen Schmitt

**Affiliations:** 1 InGef - Institute for Applied Health Research Berlin GmbH, Berlin, Germany; 2 Center for Evidence-Based Healthcare (ZEGV), University Hospital and Faculty of Medicine Carl Gustav Carus, TU Dresden, Dresden, Germany; 3 BARMER Institut für Gesundheitssystemforschung (bifg), Berlin, Germany; 4 Vandage GmbH, Bielefeld, Germany; 5 Techniker Krankenkasse, Hamburg, Germany; 6 AOK Bayern, Regensburg, Germany; 7 Robert Koch-Institut, Berlin, Germany; 8 AOK PLUS, Dresden, Germany; 9 DAK-Gesundheit, Hamburg, Germany

**Keywords:** COVID-19, Claims data, Prediction score, Vulnerable groups

## Abstract

**Aim:**

We aimed to develop a risk score to calculate a person’s individual risk for a severe COVID-19 course (POINTED score) to support prioritization of especially vulnerable patients for a (booster) vaccination.

**Subject and methods:**

This cohort study was based on German claims data and included 623,363 individuals with a COVID-19 diagnosis in 2020. The outcome was COVID-19 related treatment in an intensive care unit, mechanical ventilation, or death after a COVID-19 infection. Data were split into a training and a test sample. Poisson regression models with robust standard errors including 35 predefined risk factors were calculated. Coefficients were rescaled with a min–max normalization to derive numeric score values between 0 and 20 for each risk factor. The scores’ discriminatory ability was evaluated by calculating the area under the curve (AUC).

**Results:**

Besides age, down syndrome and hematologic cancer with therapy, immunosuppressive therapy, and other neurological conditions were the risk factors with the highest risk for a severe COVID-19 course. The AUC of the POINTED score was 0.889, indicating very good predictive validity.

**Conclusion:**

The POINTED score is a valid tool to calculate a person’s risk for a severe COVID-19 course.

**Supplementary Information:**

The online version contains supplementary material available at 10.1007/s10389-023-01884-7.

## Introduction

In the current COVID-19 pandemic, vaccinations are an essential tool to prevent severe disease courses of a SARS-Cov-2 infection and protect public health. Many countries including Germany prioritized elderly people, residents, and personnel of long-term care facilities, healthcare workers, social care personnel, and people with certain comorbidities for a COVID-19 vaccination at the beginning of the vaccination campaign due to limited vaccine availability and later on for booster vaccinations (BMG 2021; STIKO 2021). The aim of the German vaccination campaign against COVID-19 was to minimize the number of severe disease courses and COVID-19 associated mortality. Furthermore, securing the operability of the health care system and the protection of individuals at high risk of infection due to their profession (Waize et al. 2021). In Europe, vaccination campaigns against COVID-19 are advanced, but complete vaccination coverage including a booster dose varies between countries (ECDC 2022). As of 20 February 2022, 56.3% of the German population had received two doses plus a booster immunization against COVID-19 (BMG 2022). In February 2022, the *German Standing Committee on vaccination* [(STIKO [Ständige Impfkommission]) recommended a second booster vaccination for (i) people 70 years and older, (ii) residents and personnel of long-term care facilities, (iii) people with an immunodeficiency from the age of five, and (iv) employees of medical facilities, especially those with direct patient contact (STIKO 2022).

The relevance of different comorbidities as risk factors for a severe disease course of COVID-19 has been well described (Dreher et al. 2020; Gagiannis et al. 2020; Grunert et al. 2020; Härter et al. 2020; Monika et al. 2020; Nachtigall et al. 2020; Rößler et al. 2021). Relevant comorbidities include but are not limited to autoimmune diseases, (hemato-) oncological conditions as well as cardiovascular diseases such as heart failure and coronary heart disease. Limited evidence was available regarding the effect of risk factors in different age groups (Treskova-Schwarzbach et al. 2021) and whether a cumulative effect of multiple risk factors in a person is relevant regarding a persons’ risk for a severe disease course. Hence, a potentially cumulative effect of the simultaneous presence of multiple risk factors were not incorporated in the vaccination recommendations in Germany. We aimed to develop and validate a risk score to calculate a person’s individual risk for a severe COVID-19 course, which accounts for the presence of multiple risk factors at the same time and incorporates differential effects of underlying comorbidities in different age groups, using German claims data. This score (POINTED score) can be used to identify people who would most benefit from additional (personal) protective measures against COVID-19 and fourth vaccination to prevent severe courses of disease.

## Methods

### Data base

This cohort study was based on nationwide claims data of approximately 38 million individuals under statutory health insurance (SHI) in Germany. Data from two German Local Health Care Funds [AOK PLUS Sachsen and AOK Bayern], the BARMER, and the DAK-Gesundheit and the Techniker Krankenkasse (TK) as well as the research database of the Institute of Applied Health Research Berlin including anonymized claims data of company health insurances [Betriebskrankenkasse (BKK)] were used for the purposes of this analysis. Claims data from the AOK PLUS Sachsen and from DAK-Gesundheit were analyzed at the Center for Evidence-Based Healthcare (ZEGV) at the TU Dresden and Vandage GmbH, respectively. In total, the data included information of approximately 38 million persons, which corresponds to approximately 46% of the German population.

In addition to sociodemographic information (age and sex) and vital status (i.e., date of death), German claims data contain information about performed ambulatory services (according to “Einheitlicher Bewertungsmassstab,” EBM), diagnoses documented in the ambulatory and hospital setting (according to the International Statistical Classification of Diseases and Related Health Problems - German Modification, ICD-10-GM) and procedures conducted (according to the “Operationen- und Prozedurenschluessel,” OPS; German modification of the International Classification of Procedures in Medicine, ICPM) as well as drug prescription data (according to the German Anatomical Therapeutic Chemical (ATC) Classification). Longitudinally linked data from the years 2019 and 2020 have been used for the purposes of this study. Due to German data protection law, pooling of individual-level data was not feasible. Hence, six harmonized health insurance data sets were analyzed separately by authorized institutes or the healthcare research department within the respective health insurance.

### Study population

Adult patients with a confirmed ambulatory and hospital COVID-19 diagnosis (ICD-10 U07.1!; laboratory confirmed SARS-CoV-2 virus) between 27 January 2020 and 31 December 2020 were included in the analysis. COVID-19 patients had to be continuously enrolled in the SHI in the year 2019 up to the date of the COVID-19 infection and from their COVID-19 infection until death or 31 December 2020, whichever came first. Based on COVID-19 related ambulatory and hospital services the beginning of the COVID-19 infection was determined. Claims data from the year 2019 were used to assess risk factors for a severe course of COVID-19.

### Outcome

The outcome severe COVID-19 course was defined as COVID-19 related treatment in an intensive care unit, mechanical ventilation, or death after a COVID-19 infection. Intensive care treatment and mechanical ventilation had to occur within a hospitalization for which COVID-19 was documented as a discharge diagnosis. Deaths occurring within 30 days after a COVID-19 infection, during a COVID-19 related hospitalization, or within 14 days following such hospitalizations were defined as COVID-19 related. An overview of the procedure codes used to identify intensive care treatment and mechanical ventilation is provided in the supplementary material S1.

### Risk factors

Based on an umbrella review by Treskova-Schwarzbach et al. (20) and further the recommendations of the STIKO, we defined 35 conditions that were associated with a severe course of COVID-19. The conditions were defined using ICD-10 codes derived from hospital discharges and ambulatory physicians and psychotherapists in the year prior to the COVID-19 infection. Ambulatory diagnoses had to be documented in at least two quarters in 2019. Furthermore, prescriptions for specific therapies were required to validate ambulatory diagnoses of asthma, coronary heart disease, COPD, depression, diabetes, hematologic, metastatic, and solid cancer with therapy, heart failure, hypertension, and severe psychiatric diseases. The definition of risk factors is available from the corresponding author upon request.

### Statistical modeling

Statistics for the entire COVID-19 cohort across all data sites were summarized descriptively. To develop the score, the total cohorts of COVID-19 patients in 2020 selected at each data site were split into training and a test data set. The training data sets, used to develop the score, included a random 90% sample of the total study population at the respective data site. The random test data set included the other 10% of the study population and was used to assess the performance of the developed score.

To evaluate whether effect modification of certain risk factors by age was present, age stratified Poisson regression models with robust standard errors using the training data sets were estimated (Zou 2004). To estimate the model, the fisher scoring algorithm was used. Poisson regression yields consistent estimators of model coefficients irrespective of the distribution of the outcome (Gourieroux et al. 1984). The following age groups were chosen for age stratification: 18–64 years, 65 to 79 years, and 80 and older. The regression results estimated at the individual data sites were pooled with a meta-analysis using the metagen routine in the R package meta (Schwarzer 2021). The pooled age stratified results were reviewed by an expert panel of physicians to decide which age and risk factor interactions to include in the final model.

The models with the selected interaction terms were fitted at each data site and pooled again using meta-analysis. These final coefficients were rescaled with a min–max normalization on the scale 0 to 20 (Patro and Sahu 2015). Negative coefficients were set to zero.

To evaluate the scores’ discriminatory ability, the test data sets at each data site were used to determine the area under the curve (AUC) of the receiver operating characteristics (ROC) and the Youden index, the point of the ROC curve with the highest combined true positive rate (TPR) and false positive rate (FPR). The performance of the pooled score model was evaluated separately at each site. Aggregated statistics about the grouped age and score distribution and prediction performance (area under the curve) were pooled at ZEGV. The performance of the grouped risk score model was compared to the performance of a score model based on grouped age only.

## Results

A total of 623,363 patients with a confirmed COVID-19 infection between 27 January 2020 and 31 December 2020 were included in the analysis at all participating data sites. Approximately 42% were male and 22% were above the age of 64. Table 1 shows descriptive baseline characteristics for the training and the test data set. The most common risk factors included hypertension (21.68%), depression (7.09%), and chronic renal failure (7.09%) in the training data set. Due to data protection reasons, conditions that occurred in less than five patients in one of the included data sets could not be reported. Approximately 5% (n = 3297) of COVID-19 patients experienced a severe course of the COVID-19 disease (intensive care treatment, mechanical ventilation, or death after COVID-19 infection). There was no evidence for systematic differences regarding the assessed baseline characteristics or outcome frequency between training and test data set.

**Table 1:** Description of the study population of patients with COVID-19 in 2020

	90% training sample		10% test sample	
Factor of interest	N	Proportion^1^	N	Proportion^1^
Total	561,027	100%	62,336	100%
Severe disease courses of COVID-19	28,788	5.13%	3297	5.29%
Male sex	236,847	42.22%	26,466	42.46%
Age 18–24 years	54,472	9.71%	6071	9.74%
Age 25–39 years	136,055	24.25%	14,949	23.98%
Age 40–49 years	90,860	16.20%	10,010	16.06%
Age 50–54 years	56,783	10.12%	6275	10.07%
Age 55–59 years	57,105	10.18%	6531	10.48%
Age 60–64 years	40,930	7.30%	4638	7.44%
Age 65–69 years	22,711	4.05%	2526	4.05%
Age 70–74 years	18,872	3.36%	2144	3.44%
Age 75–79 years	20,864	3.72%	2239	3.59%
Age 80 years and older	62,375	11.12%	6953	11.15%
Asthma	13,706	2.44%	1465	2.35%
Asthma and 18–64 years	9262	2.12%	960	1.98%
Asthma and 65 years and older	4444	3.56%	505	3.64%
Arrhythmia or Atrial fibrillation	29,190	5.20%	3252	5.22%
Autoimmune diseases	38,337	6.83%	4373	7.02%
Cerebrovascular diseases	34,468	6.14%	3755	6.02%
Cerebrovascular diseases (incl. stroke) and 18–79 years	17,207	3.45%	1865	3.37%
Cerebrovascular diseases (incl. stroke) and 80 years and older	17,261	27.67%	1890	27.18%
Chronic obstructive pulmonary disease (COPD)	15,955	2.84%	1775	2.85%
Chronic renal insufficiency	39,747	7.08%	4444	7.13%
Chronic renal insufficiency and 18–79 years	17,728	3.56%	1955	3.53%
Chronic renal insufficiency and 80 or older	22,019	35.30%	2489	35.80%
Cirrhotic and severe liver disease	2617	0.47%	284	0.46%
Coronary heart disease	37,282	6.65%	4118	6.61%
Crohn’s disease	2290	0.41%	277	0.44%
Dementia	29,072	5.18%	**	**
Dementia and 18–64 years	962	0.22%	*	*
Dementia and 65 years and older	28,110	2.25%	3142	2.67%
Depression	39,750	7.09%	4393	7.05%
Depression and 18–79 years	30,270	6.07%	3328	6.01%
Depression and 80 and older	9480	15.20%	1065	15.32%
Diabetes type I and II	39,998	7.13%	4291	6.88%
Diabetes type I and II and 18–64 years	13,440	3.08%	1453	3.00%
Diabetes type I and II and 65–79 years	12,376	19.82%	1378	19.94%
Diabetes type I and II and 80 and older	13,182	21.13%	1460	21.00%
Dialysis	2550	0.45%	**	**
Dialysis and 18–64 and older	777	0.18%	*	*
Dialysis and 65 years and older	1773	1.42%	201	1.45%
Down syndrome	570	0.10%	*	*
Heart failure	32,467	5.79%	3566	5.72%
Hematologic oncologic conditions without therapy	2839	0.51%	310	0.50%
Hematologic oncologic conditions with therapy	909	0.16%	**	**
Hematologic oncologic conditions with therapy and 18–64 years	328	0.08%	*	*
Hematologic oncologic conditions with therapy and 65 years and older	581	0.47%	78	0.56%
Hepatitis	1452	0.26%	152	0.24%
HIV	775	0.14%	*	*
Hypertension	121,621	21.68%	13,546	21.73%
Hypertension and 18–79 years	79,000	15.84%	8827	15.94%
Hypertension and 80 years and older	42,621	68.33%	4719	67.87%
Immunocompromising conditions	13,315	2.37%	1517	2.43%
Immunosuppressive therapy	8570	1.53%	955	1.53%
Immunosuppressive therapy and 18–64 years	4939	1.13%	546	1.13%
Immunosuppressive therapy and 65 years and older	3631	2.91%	409	2.95%
Impairment of intelligence	4456	0.79%	496	0.80%
Innate Immunodeficiency	1164	0.21%	120	0.19%
Interstitial lung disease	1122	0.20%	123	0.20%
Metastasized solid cancer without therapy	2022	0.36%	219	0.35%
Metastasized solid cancer with therapy	1827	0.33%	227	0.36%
Neurologic diseases	33,467	5.97%	3717	5.96%
Neurologic diseases (incl. Morbus Parkinson, epilepsy) and 18–64 years	11,276	2.59%	1231	2.54%
Neurologic diseases (incl. Morbus Parkinson, epilepsy) and 65–79 years	8929	14.30%	1007	14.58%
Neurologic diseases (incl. Morbus Parkinson, epilepsy) and 80 years and older	13,262	21.26%	1479	21.27%
Obesity	19,295	3.44%	2124	3.41%
Obesity and 18–64 years	12,892	2.96%	1396	2.88%
Obesity and 65–79 years	4150	6.65%	464	6.72%
Obesity and 80 and older	2253	3.61%	264	3.80%
Rheumatic diseases	16,863	3.01%	1881	3.02%
Severe psychiatric diseases (incl. Schizophrenia)	4915	0.88%	547	0.88%
Solid cancer without therapy	25,206	4.49%	2775	4.45%
Solid cancer with therapy	4156	0.74%	486	0.78%
Status post organ transplantation	996	0.18%	122	0.20%
Ulcerative colitis	2785	0.50%	345	0.55%

*Due to sample sizes below five in a least one of the six data sets this number is not reported.

**Due to sample sizes below five in one of the disease-age-strata a total is not reported.

^1^Proportions relative to the total number of patients with COVID-19 in the data set/in the respective age group

The min–max normalized POINTED score values as well as the risk ratios derived from the pooled Poisson regression results are displayed in Table 2. Due to the reference category “18 to 24 years,” the exponentiated coefficients for the age groups appear high compared to the disease/no disease ratios. The factors with the highest risk for a severe course of COVID-19 besides age included down syndrome (6 points) and hematologic cancer with therapy in patients between 18 and 64 years (5 points), immunosuppressive therapy in patients between 18 and 64 years (4 points), and other neurological conditions in that same age group (4 points). No excess risk was found for ulcerative colitis, Crohn’s disease, rheumatic diseases, and dialysis in patients over the age of 64 years and depression in patients 80 years and older. A patient’s individual risk score can be calculated by adding the score across all disease categories this patient suffers from. The total score for an exemplary 66-year-old (16 points), male (3 points) patient with COPD (1 point), and heart failure (1 point) is 21 points.

**Table 2:** Rounded estimates of the POINTED risk score for severe courses of COVID-19

Factor	POINTED risk score	Risk ratio (95% CI)
Age 18–24	0	1 (Ref.)
Age 25–39	3	2.021(1.587– 2.573)
Age 40–49	7	4.979(3.874–6.400)
Age 50–54	9	7.842(6.018–10.219)
Age 55–59	11	11.032(8.766–13.884)
Age 60–64	12	16.629 (13.307–20.781)
Age 65–69	16	39.169 (31.280–49.049)
Age 70–74	17	53.396 (42.855–66.529)
Age 75–79	18	64.531 (51.804–80.384)
Age 80+	20	120.067 (96.608–149.222)
Male sex	3	1.760(1.667–1.857)
Arrhythmia or atrial fibrillation	1	1.072(1.018–1.130)
Asthma 18–64	1	1.146(0.931–1.410)
Asthma 65+	0	0.864(0.796–0.937)
Autoimmune diseases	0	0.981(0.948–1.015)
Coronary heart disease	1	1.036(1.009–1.064)
Cerebrovascular diseases (incl. stroke) 18–79	1	1.102(1.055–1.151)
Cerebrovascular diseases (incl. stroke) 80+	1	1.004(0.967–1.042)
Chronic renal insufficiency 18–79	2	1.413(1.355–1.473)
Chronic renal insufficiency 80+	1	1.149(1.116–1.184)
Ulcerative colitis	0	0.957(0.841–1.089)
Chronic obstructive pulmonary disease (COPD)	1	1.164(1.124–1.205)
Dementia 18–64	2	1.583(1.224–2.047)
Dementia 65+	1	1.193(1.116–1.277)
Depression 18–79	1	1.079(1.029–1.132)
Depression 80+	0	0.977(0.924–1.032)
Diabetes type I and II 18–64	3	1.905(1.597–2.271)
Diabetes type I and II 65–79	2	1.289(1.232–1.350)
Diabetes type I and II 80+	1	1.115(1.081–1.151)
Dialysis 18–64	3	2.000(1.286–3.109)
Dialysis 65+	0	0.892(0.745–1.068)
Down syndrome	6	3.884(2.927–5.154)
Hematologic oncologic conditions without therapy	2	1.313(1.202–1.434)
Hematologic oncologic conditions with therapy 18–64	5	2.997(2.163–4.153)
Hematologic oncologic conditions with therapy 65+	2	1.496(1.302–1.719)
Hepatitis	1	1.060(0.898–1.251)
Heart failure	1	1.210(1.127–1.299)
HIV	2	1.435(1.028–2.003)
Immunocompromising conditions	1	1.189(1.108–1.276)
Immunosuppressive therapy 18–64	4	2.285(2.026–2.576)
Immunosuppressive therapy 65+	2	1.294(1.227–1.366)
Innate immunodeficiency	1	1.002(0.761–1.321)
Interstitial lung disease	2	1.326(1.205–1.460)
Impairment of intelligence	2	1.318(1.153–1.506)
Metastasized solid cancer without therapy	2	1.501(1.299–1.733)
Metastasized solid cancer with therapy	2	1.610(1.309–1.980)
Crohn’s disease	0	0.912(0.735–1.131)
Status post organ transplantation	2	1.301(1.150–1.473)
Neurologic diseases 18–64	4	2.110(1.833–2.429)
Neurologic diseases 65–79	1	1.179(1.124–1.238)
Neurologic diseases 80+	1	1.016(0.983–1.051)
Severe psychiatric diseases (incl. Schizophrenia)	1	1.204(1.122–1.293)
Rheumatic diseases	0	0.992(0.948–1.038)
Cirrhotic and severe liver disease	2	1.417(1.166–1.720)
Solid cancer without therapy	1	1.001(0.954–1.051)
Solid cancer with therapy	1	1.180(1.089–1.279)
Hypertension 18–79	2	1.282(1.224–1.343)
Hypertension 80+	1	1.013(0.946–1.084)
Obesity 18–64	3	1.706(1.546–1.884)
Obesity 65–79	1	1.237(1.165–1.314)
Obesity 80+	1	1.044(0.979–1.114)

Factors that increase the risk of a severe COVID-19 disease course were more prevalent in the higher age groups (see Table 1). In the training data set, 1.1% of COVID-19 patients between 18 and 64 years received an immunosuppressive therapy compared to 2.9% of COVID-19 patients 65 years and older. However, the effect of certain risk factors was more pronounced in the younger age groups. The risk score for patients under immunosuppressive therapy between 18 to 64 years is 4, while the score is 2 in patients above 65 years of age. The same was observed for type I and II diabetes. The additional risk for a severe disease course of COVID-19 is highest in diabetic patients between 18 to 64 years and lower in the older age groups, while the disease is more common in the older age group.

**Fig. 1:**
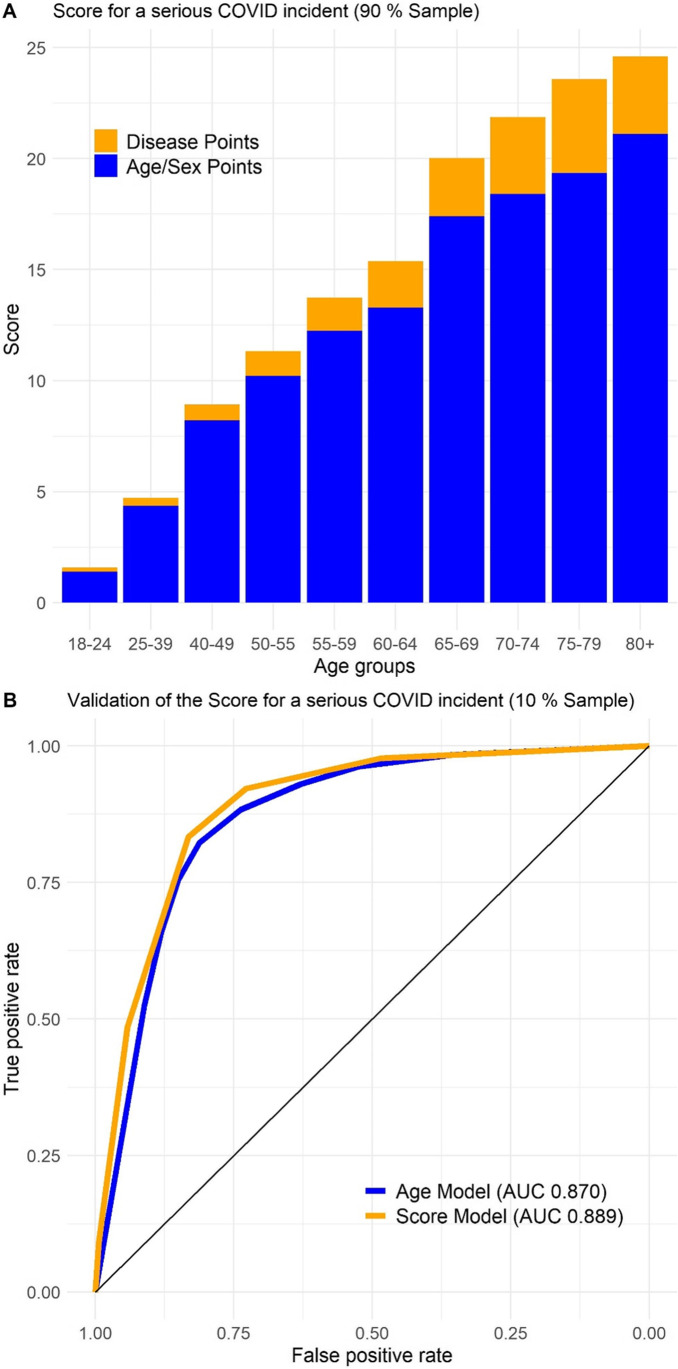
**A** Comparison of the POINTED risk score and the score based on age and sex over the different age groups for the 90% training sample. **B** ROC curve and AUC for age model and POINTED score model in the 10% test data

To quantify the additional value of incorporating comorbidities and sex in the score compared to an age and sex-based score only, we compared the mean score in each age group based on the age/sex group scores and the mean scores using age/sex group and disease scores (see Fig. 1A). The POINTED risk score (orange) is higher than the score using only the estimated pointes for age and sex (blue). Especially in the higher age groups, incorporating the added risk of certain diseases leads to higher average POINTED scores.

### Validation of the score

The AUC for the prediction of severe COVID-19 courses was similar when using the developed POINTED risk score (0.889) or a score based on age alone (0.870) (see Fig. 1B).

The distribution of the severe courses of COVID-19 by age group and groups of the POINTED risk score is depicted in Table 3. Only 0.24% of all COVID-19 patients in the age group 18 to 39 years developed a severe course of COVID-19, but 24.7% of those with 80 or more years of age. In patients with a POINTED risk score below 10, 0.26% experience a severe COVID-19 disease course compared to 43.6% of the patients with a score of 30 and more (Table 3).

**Table 3:** Number of patients with and without a severe course of COVID-19 in the test data set, stratified by age group and points of the POINTED risk score

	Absolute number of patients	
Age group	with a severe COVID-19 course	without a severe COVID-19 course
18–39 years	51	20,969
40–49 years	78	9932
50–54 years	105	6170
55–59 years	148	6383
60–64 years	208	4430
65–69 years	228	2298
70–74 years	321	1823
75–79 years	439	1800
80 years and older	1719	5234
POINTED risk score		
0–9 points	75	28,484
10–14 points	177	14,481
15–19 points	298	6153
20–24 points	1149	6459
25–29 points	1292	3066
30 + points	306	396

Based on the Youden Index two cut-off points were defined: 65 years and older (TPR 0.823; FPR 0.812) for the age score and 20 points and more (TPR 0.833; FPR 0.832) for the POINTED risk score including sex and comorbidities as risk factors. Given that a vaccination would prevent 100% of severe courses of COVID-19 in people 65 years and older in the test data (n = 13,862), 2707 cases of severe COVID-19 cases could have been prevented (see Table 3) in that age group. Due to the relative rareness of the outcome of about 5%, this corresponds to a positive predictive value (PPV) of 0.195 (2707/13,862), i.e., about one fifth of the patients who have been identified to be at high risk for a severe course of COVID-19 because they are 65 years or older experienced the outcome. The PPV increases to 0.217 (2747/12,668) when patients with a POINTED risk score of 20 or more points are prioritized for a vaccination.

## Discussion

We derived and internally validated a risk score (POINTED score) for a severe COVID-19 disease course in a population of 623,363 COVID-19 patients in Germany which aimed to optimize prioritization for a COVID-19 vaccination by considering the potentially cumulative effect of different comorbidities. The score adequately predicted a severe course of COVID-19 (AUC 0.889) in a validation cohort of 62,336 German COVID-19 patients. Using the presented methodology individuals can be prioritized for vaccination in descending order of the estimated risk score per person. The additive score allows for the consideration of multiple risk factors since individuals with multiple low risk conditions might have an equal risk for a severe COVID-19 course as patients with one major risk factor only. The POINTED score performed only slightly better than a model based on age only in prediction of a severe course of COVID-19. This underlines that a risk stratification by age alone is also a feasible way for prioritization. However, younger patients with major chronic diseases benefit from the POINTED risk score as they would qualify for an earlier vaccination than when only age is used for prioritization. On the other hand, older patients with an age below 80 and no or only minor documented chronic diseases would eventually have to wait longer. Furthermore, the score can be used for prioritization within age groups.

Jucknewitz et al. also identified and quantified risk factors for a severe course of COVID-19 (Jucknewitz et al. 2022). The authors included prediction variables in a more granular level and refrained from grouping of ICD-10, ATC, or procedure codes to predefined potential risk factors to avoid the loss of information. In contrast, we chose to define a set of risk factors, which had been shown to be associated with a higher risk for a severe course of COVID-19 (Treskova-Schwarzbach et al. 2021). This allows for an easier interpretation and application of the results by medical professionals.

In contrast to an earlier work (Wende et al. 2022), we decided to develop a score in a cohort of COVID-19 patients instead of the general population. Hence, our results are not confounded by different probabilities of contracting the disease within the populations with risk factors (e.g., strict self-isolation) as we only assess the impact of risk factors on severe course of COVID-19 once infected. For identifying people who would benefit most from a vaccination in the overall population, we think that this is the adequate approach. In line with our results, a British study using a database comprising general practices in England with linkage to Covid-19 test results, Hospital Episode Statistics, and death registry found that down syndrome and dementia significantly increased the risk for a severe course of COVID-19 (Clift et al. 2020). Of all considered factors, only asthma in the age group 65+ was associated with a significant lower likelihood of a severe course of COVID-19. This might be due to the specific medication not only controlling the chronic disease but also being beneficial during an acute COVID-19 infection (Izquierdo et al. 2021).

### Limitations

Most importantly, the presented score was developed in an unvaccinated population and the risk of a severe course if COVID-19 is lower in vaccinated individuals. However, as risk factors for a severe disease course are similar in vaccinated and unvaccinated individuals, we consider the developed score to be valid in a vaccinated population also (Antonelli et al. 2022; Yek et al. 2022). Accordingly, the STIKO recommendation for a fourth vaccination for especially vulnerable or exposed groups is also based on the previously established risk factors for a severe course of COVID-19 (STIKO 2022).

Due to data protection regulations, we had to use meta-analytic methods to pool the results of the individual data holders. This causes a loss in efficiency compared to direct estimation.

Furthermore, German claims data, especially data from the outpatient setting, are only available with a time delay. Hence, for this paper we could only include data of COVID-19 patients until 31 December 2020. Consequently, no COVID-19 infections with variants such as Delta or Omicron were included in the analysis. However, if the risk factors for a severe course for COVID-19 were similar between the variants, the results of this study are still applicable.

The developed score will be most feasible for application in populations with a similar burden of disease of the considered risk factors. However, using the described methodology the score can be used or rapidly adapted to specific populations given that an adequate population-based database for the calculation of the score is available. The methodological approach is transferable to other situations where the cumulative effect of multiple risk factors is to be estimated for a risk ranking in a defined patient population.

### Conclusion

The presented POINTED score offers an opportunity for physicians and all healthcare decision makers (e.g., health insurance companies, German National Association of Statutory Health Insurance Physicians) to calculate a person’s individual risk for a severe course of COVID-19. This supports the prioritization of especially vulnerable patients for booster vaccinations or other protective public health measures to prevent severe courses of disease.

## Supplementary information

ESM 1 (DOC 23 kb)

## Data Availability

As described in the methods section, German data protection laws do not allow a pooling of claims data from different statutory health insurances without prior regulatory approval, which can take up to nine months. Considering the dynamic situation during the pandemic, we chose to pool aggregate data instead. The raw data used in this study cannot be made available in the manuscript, the supplemental files, or in a public repository due to German data protection laws (Bundesdatenschutzgesetz). The aggregated data is stored on a secure drive at ZEGV.

## Code availability

The R code of the analysis can be made available upon request by the corresponding author.
