# Supplementary material for: Development of a risk score to identify patients at high risk for a severe course of COVID-19
Source: Z Gesundh Wiss. 2023 Mar 22:1–10. Online ahead of print. doi: 10.1007/s10389-023-01884-7 (PMC10032626; doi:10.1007/s10389-023-01884-7)
Supplement: Supplementary file 1 — (DOC 23 kb) [file 10389_2023_1884_MOESM1_ESM.doc]

**S1 Procedure Codes [German Operationen- und Prozedurenschlüssel (OPS)] used for identification of intensive care treatment and mechanical ventilation (German only)**

8-701 Einfache endotracheale Intubation

8-704 Intubation mit Doppellumentubus

8-706 Anlegen einer Maske zur maschinellen Beatmung

8-712 Maschinelle Beatmung und Atemunterstützung bei Kindern und Jugendlichen

8-714 Spezialverfahren zur maschinellen Beatmung bei schwerem Atemversagen

8-97a Multimodale intensivmedizinische Überwachung und Behandlung bei zerebrovaskulären Vasospasmen

8-97b Multimodale intensivmedizinische Überwachung und Behandlung bei neuromuskulären Erkrankungen

8-980 Intensivmedizinische Komplexbehandlung (Basisprozedur)

8-98d Intensivmedizinische Komplexbehandlung im Kindesalter (Basisprozedur)

8-98f Aufwendige intensivmedizinische Komplexbehandlung (Basisprozedur)

8-920 EEG-Monitoring

8-921 Monitoring mittels evozierter Potenziale

8-923 Monitoring der hirnvenösen Sauerstoffsättigung

8-924 Invasives neurologisches Monitoring

8-930 Monitoring von Atmung, Herz und Kreislauf I

8-931 Monitoring von Atmung, Herz und Kreislauf II

8-932 Monitoring von Atmung, Herz und Kreislauf III
